# Microbiome and Exudates of the Root and Rhizosphere of *Brachypodium distachyon*, a Model for Wheat

**DOI:** 10.1371/journal.pone.0164533

**Published:** 2016-10-11

**Authors:** Akitomo Kawasaki, Suzanne Donn, Peter R. Ryan, Ulrike Mathesius, Rosangela Devilla, Amanda Jones, Michelle Watt

**Affiliations:** 1 CSIRO Agriculture and Food, Canberra, ACT, Australia; 2 Hawkesbury Institute for the Environment, Western Sydney University, Richmond, NSW, Australia; 3 Division of Plant Science, Research School of Biology, Australian National University, ACT, Australia; 4 Institute of Bio and Geosciences (IBG 2), Plant Sciences, Forschungszentrum Jülich GmbH, Jülich, Germany; Montana State University Bozeman, UNITED STATES

## Abstract

The rhizosphere microbiome is regulated by plant genotype, root exudates and environment. There is substantial interest in breeding and managing crops that host root microbial communities that increase productivity. The eudicot model species Arabidopsis has been used to investigate these processes, however a model for monocotyledons is also required. We characterized the rhizosphere microbiome and root exudates of *Brachypodium distachyon*, to develop it as a rhizosphere model for cereal species like wheat. The Brachypodium rhizosphere microbial community was dominated by Burkholderiales. However, these communities were also dependent on how tightly they were bound to roots, the root type they were associated with (nodal or seminal roots), and their location along the roots. Moreover, the functional gene categories detected in microorganisms isolated from around root tips differed from those isolated from bases of roots. The Brachypodium rhizosphere microbiota and root exudate profiles were similar to those reported for wheat rhizospheres, and different to Arabidopsis. The differences in root system development and cell wall chemistry between monocotyledons and eudicots may also influence the microorganism composition of these major plant types. Brachypodium is a promising model for investigating the microbiome of wheat.

## Introduction

*Brachypodium distachyon* was proposed as a model species for the Pooideae family in 2001 because of its small stature, rapid life cycle, and small genome size of 272 Mb [1]. *B*. *distachyon* and other Brachypodium species are now important tools for investigating grasses because the growing availability of genetic resources include a fully sequenced genome, a large collection of accessions [2] and T-DNA mutants [3]. Brachypodium serves as a functional genomics model in elucidating cereal genomes [4] as well as for studying small noncoding RNAs such as microRNAs [5, 6]. This species has also been studied for flowering time variation [7], plant-pathogen relations [8–10], plant microbe relations [11, 12], and for root architecture and genetics [13–15]. Brachypodium provides a convenient model for studying cereal root systems because its mature roots are less than a third of the size of cereal crops such as wheat, maize and rice, and therefore are more amenable to laboratory and glasshouse studies [14]. This paper reports on the characterization of the root microbiome and exudates of Brachypodium to validate their role as a model for rhizosphere biology in cereal crops.

Rhizosphere biology can influence the productivity of plants [16, 17]. Rhizosphere microorganisms benefit plant growth by increasing nutrient supply to plants, suppressing pathogens, and by carrying out other less studied roles [18]. Plant growth promoting (PGP) strains of *Azospirillum* and *Herbaspirillum* have been reported to colonize Brachypodium roots and enhance growth of some Brachypodium genotypes under low or no nitrogen conditions [11]. Inoculation with the PGP strain *Bacillus subtilis* B26 increased Brachypodium biomass and also enhanced plant drought resistance [12].

Plants release between 5 and 25% of net fixed carbon into the rhizosphere in the form of compounds ranging from simple organic anions to complex polymer mucilages [19]. Such root exudates can alter the rhizosphere microbial community structure and diversity compared to the bulk soil, and each plant species harbours a set of specific rhizosphere microbial populations due, in part, to differences in composition of the root exudates [20, 21]. Root exudation is also influenced by various biotic and abiotic factors in the surrounding environment, which can lead to a significant shift in the rhizosphere microbiota [22–25].

There is a requirement to understand the plant-soil interface sufficiently well to allow the rhizosphere to be engineered to benefit plant fitness in cereals [16, 26–28]. An important step is the development of robust plant models for this complex system. Characterizing the core microbial communities in the rhizosphere and identifying the major root exudates are critical inputs to such models. This information is now being collected in model plants such as Arabidopsis [29, 30] and in crop species such as wheat [31, 32], rice [33], and maize [34]. A recent study used Arabidopsis, Brachypodium and Medicago to investigate shifts in the microbial populations in the soil over successive plantings, and the three models modified the soil microbiomes differently [35].

We hypothesized that the root microbiome and root exudates of Brachypodium would be more similar to wheat than to the model eudicot Arabidopsis, as cereals develop different types of roots (primary seminal and nodal root systems, each with branch roots), while eudicots develop a single taproot system. We characterized the core bacterial and fungal communities in the Brachypodium rhizosphere on two different root types (seminal and nodal), and examined the effects of distance from root and root attachment on these populations. We also catalogued the major organic exudate compounds and compared all these findings with other species. This study helps to establish Brachypodium as a model species for studying the rhizosphere biology of cereal crops.

## Materials and Methods

Three experiments were conducted. Experiment 1 describes the microbiome of the Brachypodium rhizosphere, and how this is influenced by distance and attachment to the roots. Experiment 2 tested if primary seminal and leaf nodal roots generate different microbial communities. Experiment 3 characterised the major root exudates from Brachypodium since these compounds can influence the composition of the rhizosphere microbiome.

### Soil and plant growth conditions

Agricultural soil (0–20 cm depth) was collected from CSIRO Ginninderra Experiment Station (Canberra, ACT, Australia; 35°20’17”S, 149°07’96”E). It is a shallow red-yellow podzolic soil with pH of 4.91 [36]. Soil was sieved (2 mm), air dried and stored at room temperature before mixing with autoclaved river sand (20% soil/80% sand, v/v) for Experiments 1 and 2.

*Brachypodium distachyon* Bd21-3 was used for all experiments because genetic resources and a large number of T-DNA mutants are available in this accession [3]. Original seeds were sourced from Dr John Vogel (DOE Joint Genome Institute, CA). For all experiments, seeds were dehusked and surface-sterilized with chlorine gas fumigation for 1 hour, followed by washing with 3% (w/v) sodium hypochlorite for 20 min. After sterilization, seeds were rinsed multiple times with sterile water. For Experiment 1, sterilized seeds were pre-germinated on 2% water agar at 25°C for 3 days in the dark. Two seedlings were transplanted into the soil/sand mix in a plastic pot (12 cm diameter, 14 cm height), and cultivated for 4 weeks in a growth cabinet (five replicates).

For Experiment 2, the soil/sand mix was packed into 25 cm tall and 9 cm diameter PVC tubes (bottoms sealed with a petri dish with drainage holes) lined with plastic bags (to easily remove plant root systems). Sterilized seeds were sown into the soil/sand mix at 2 cm depth (four seeds/tube), watered to saturation, and stratified in a cold room (6°C) for 7 days. PVC tubes were then moved to the growth cabinet and plants were cultivated for another 23 days (30 days from sowing) or 37 days (44 days from sowing) (nine replicates each). Whenever more than two seedlings emerged, they were removed and only two seedlings were kept per tube.

All experiments were carried out in a growth cabinet (Conviron, Canada) set to 16 hours day/8 hours night, 24°C day/18°C night, with a light intensity of 500 μmol m^-2^ s^-1^. Pots and tubes were topped with plastic pellets to prevent excessive moisture loss, and where required, the plants were watered with ¼ strength Hoagland’s solution [37].

For each experiment, unplanted bulk soil/sand mix samples were prepared, and treated as planted samples.

### Rhizosphere and root sampling

For Experiment 1, the Brachypodium root system was carefully removed from the pot, and attached soil gently shaken off the roots. The rhizosphere (roots and attached soil) was vortexed for 3 × 30 sec in 30 ml of sterile 0.2 mM CaCl_2_ to separate the rhizosphere into two fractions: the tightly-bound fraction (washed roots, rhizoplane and endorhizosphere) and the loosely-bound fraction (washed off rhizosphere soil, ectorhizosphere) [31]. Roots (tightly-bound fraction) were removed and stored at −80°C. Tubes with solutions were centrifuged, the supernatant removed, and the soil pellet (loosely-bound fraction) stored at −80°C. For the bulk soil sample (bulk soil/sand mix), a small amount (~2 g) of the soil was collected from the middle of the unplanted pot by digging with a spatula, and stored at −80°C before DNA extraction.

For Experiment 2, seminal and leaf nodal roots were sampled from the plants growing in the PVC tubes at day 30 and 44 after sowing. These root types were chosen because they are prominent in cereals, clearly distinguishable from other types and easy to obtain sufficient tissue from our experiments. In preliminary studies, it was confirmed that nodal roots emerge 14 days after the seminal root emergence (data not shown). Two sampling time points enabled us to compare different root types of the same age. The plant root system was removed from the PVC tube by removing the plastic lining, and gently washed to remove the sand/soil mix with running tap water. The whole plant was placed in sterile phosphate buffered saline (PBS), and seminal and nodal axile roots cut at their base and carefully detangled. Root tips and bases of seminal and nodal types (4 cm each of axiles plus branch roots) were sampled (S1 Fig), and washed by vortexing in sterile PBS. When more than one nodal root was present, the longest root was sampled. Bulk soil samples were collected from the top (1 cm from surface), middle (centre of PVC tube) and the bottom (1 cm from base) of the control unplanted tubes at day 30 and 44. Harvested samples were stored at −80°C before DNA extraction.

### DNA extraction and microbial community analysis

#### DNA extraction

DNA was isolated from soil and root samples using a PowerSoil DNA Isolation Kit (MO BIO Laboratories, Inc., Carlsbad, CA, USA) according to kit guidelines, except the bead beating step used a TissueLyser LT bead mill (QIAGEN) at 50/sec oscillation for 1 min. Prior to DNA isolation, root samples were ground and homogenized with pestle and mortar in liquid nitrogen.

#### T-RFLP analysis

Rhizosphere bacterial and fungal community structures were analyzed with terminal restriction fragment length polymorphism (T-RFLP), targeting bacterial 16S ribosomal RNA (16S rRNA) and fungal internal transcribed spacer (ITS) genes. For bacterial communities, PCR was performed with the 799F-FAM ([5’ end labelled with 6-carboxyfluorerescein] AACMGGATTAGATACCCKG) [38] and 1525R (AAGGAGGTGWTCCARCC) [39] primer set. For fungal communities, PCR was performed with the ITS1F-FAM ([5’ end labelled with 6-carboxyfluorerescein] CTTGGTCATTTAGAGGAAGTAA) [40] and ITS4 (TCCTCCGCTTATTGATATGC) [41] primer set. PCR was carried out in a 50 μl reaction with MyTaq Red DNA Polymerase (Bioline) according to the manufacturer’s protocol, and the template was 1 μl of the extracted DNA. For each sample, two PCR reactions were prepared, and the PCR products were combined and purified with SureClean Plus (Bioline).

The purified PCR products were digested with *HhaI* restriction endonuclease (New England Biolabs), and the digested DNA (2 μl, 70 ng) was mixed with 7.95 μl of HiDi formamide (Applied Biosystems) and 0.05 μl of GS600 LIZ size standard (Applied Biosystems). The terminal restriction fragments (T-RFs) were separated by capillary electrophoresis using a 3500 Genetic Analyzer (Applied Biosystems). Peak size, area and height were determined with GeneMapper software v4.0 (Applied Biosystems), and the ‘true’ peaks were selected as described previously [42]. T-RFs were then binned using a custom R script ‘interactive binner’ [43, 44] to give a matrix with relative T-RF abundance in each sample.

#### Bacterial community sequencing

Pyrosequencing was performed to characterize the rhizosphere bacterial community. Bacterial 16S rRNA genes were initially PCR-amplified with the 799F and 1525R primer set (chosen because it does not amplify Brachypodium plant-derived sequences) as described above, and the purified amplicons were amplified and sequenced on a Roche GS FLX+ platform using the 799F and 1394R (ACGGGCGGTGTGTRC) [45] primer set (Experiment 1) or 799F and 1193R (ACGCATCCCCACCTTCCTC) [29] primer set (Experiment 2) by Molecular Research LP (Shallowater, TX, USA).

Sequences were analyzed using MOTHUR v.1.32.0, following the protocol of Schloss *et al*. [46] (http://www.mothur.org/wiki/454_SOP, website accessed on July 2015). Short and low quality sequences were removed, and remaining sequences were aligned with the SILVA database (release 119) [47]. Unaligned sequences and chimeras were removed, and the valid sequences were clustered into OTUs (Operational Taxonomic Units) at 97% similarity. Sequences were then randomly subsampled from each sample (1015 sequences for Experiment 1 and 3534 sequences for Experiment 2) to achieve an even sequencing depth, and the taxonomy was assigned to OTUs by comparing to the RDP trainset ver. 14 [48].

#### Fungal community sequencing

The rhizosphere fungal community was characterized by sequencing the ITS (Internal Transcribed Spacer) region with the ITS1F and ITS4 primer set using Illumina MiSeq system by Molecular Research LP (Shallowater, TX, USA). Fungal ITS sequencing data were analyzed based on the pipeline described by Balint *et al*. [49]. Low quality sequences were removed, and the ITS1 sequences were extracted with FungalITSextractor [50]. Similarity clustering and OTU identification of the extracted ITS1 sequences was done based on the UPARSE pipeline [51] of the USEARCH v7.0.1090 [52]. Reference-based chimera filtering was performed with USEARCH v7.0.1090 [52], with UNITE fungal ITS reference dataset (version 6 for UCHIME, released on 26 July 2014) [53] as the reference. In order to achieve equal sequence depth, 49096 sequences were subsampled from each sample, and taxonomic assignment of the valid OTUs was performed by BLAST searching against the UNITE ITS database (version 6, released on 10 September 2014) using QIIME 1.6.0 [54] implemented in the Galaxy service [55] provided by the CSIRO Bioinformatics Core and IM&T (http://galaxy.bioinformatics.csiro.au/).

#### Prediction of bacterial functional gene content

Functional gene content of the rhizosphere bacterial community was predicted from the 16S pyrosequencing data using PICRUSt (phylogenetic investigation of communities by reconstruction of unobserved states) [56]. The Greengenes database (ver. 13.5) was used to assign taxonomy to the OTUs, and the functional gene content prediction was performed with the online Galaxy version of PICRUSt. The inferred gene content was hierarchically categorized up to three tiers with the KEGG Orthology (KO) database [57].

### Root exudate analysis

#### Root exudate collection

For Experiment 3, sterilized seeds were germinated in a sterile semi-hydroponic system, which consisted of a plastic tissue culture container (6.5 cm diameter × 15 cm height) filled with 2 mm glass beads (up to 5 cm height) and saturated with ¼ strength Hoagland’s solution (63 ml, pH 6.0, buffered with 2 mM MES) (S2A Fig). Seeds were placed on the bed of glass beads (30 seeds/container) and containers were incubated in a growth cabinet. On every third day, the Hoagland’s solution was agitated by gently rotating the container, and containers randomized for equal lighting. Nutrient solution was not renewed throughout the experiment, but leaf tissue mineral analysis showed that the plants were not deficient in nutrients (data not shown). Sterility of the semi-hydroponic system was confirmed by plating out the Hoagland’s solution onto LB plates before harvesting the plants.

After 3 weeks of cultivation, plants from two containers were pooled, and root exudates collected in a glass jar by immersing the plant roots in 20 ml of sterile ultra-pure water (S2B Fig). Jars were placed on an orbital shaker (60 rpm) for 3 hours in the growth cabinet, and then root exudate solution was filtered through a 0.22 μm PHENEX RC syringe filter (Phenomenex, Lane Cove, NSW, Australia). Four to nine replicates were prepared for each treatment and collected exudate solution was stored at −20°C until analysis.

#### Free Amino acid analysis

Aliquots of root exudate (10ml) were spiked with L-norleucine as the internal standard (10 μl of 0.01 mg/ml) and lyophilized. Samples were submitted to a clean-up step with cold acetone at −20°C overnight to eliminate possible protein interferences, and supernatants were transferred to new tubes and dried under vacuum. Free amino acids in the exudate samples were determined by pre-column derivatization using the AccQ∙Tag Chemistry Kit (Waters Corporation, Milford, MA, USA), according to the manufacturer. Derivatized samples were filtered through 0.2 μm polytetrafluoroethylene (PTFE) spin filters (Thermo Fisher Scientific) followed by a 10 μl injection. Free amino acids were separated in reversed-phase HPLC on an AccQ∙Tag column (4 μm, 3.9 × 150 mm, Waters) with a C18 guard column, using an Agilent 1200 Series HPLC. Analytes were eluted with a multi-step gradient of AccQ∙Tag Eluent A (Waters), acetonitrile and ultra-pure water, following AccQ∙Tag Chemistry Kit protocol, with modifications for improved separation of γ-aminobutyric acid (GABA), asparagine and norleucine. Amino acids were detected (λ_exc_ 250 nm; λ_em_ 395 nm) with an on-line fluorescence detector. Peaks were identified based on retention times of authentic standards and quantified by a linear calibration curve using Agilent ChemStation Rev. B.04.01 software.

#### Sugar analysis

Root exudates (10 ml) were lyophilized, redissolved in 40 μl of H_2_O, and filtered through a 0.2 μm PTFE spin filter (Thermo Scientific). Recovery control samples were prepared by spiking 0.06 μg of arabinose into 10 ml of ultra-pure water, lyophilized, and treated the same as the real samples.

Sugars in exudates were analyzed by anion exchange HPLC using a Dionex System equipped with a CarboPac PA20 Analytical Column (6.5 μm, 3.0 × 150 mm) and an AminoTrap guard-column (3 × 30 mm). Arabinose, galactose, glucose, fructose, sucrose and xylose were separated with a multi-step gradient of 10 mM NaOH, 200 mM NaOH and ultra-pure water. The flow rate was 0.3 ml/min, column temperature was 30°C and 20 μl of sample was injected. Sugars were detected with a Coulochem III detector (Dionex) configured with pulse-mode amperometric cell and gold electrode. Data analysis was performed using Chromeleon 6.80 SR10 software. Sugars were quantified with external standards using linear calibration curves (R^2^ = 0.99).

#### Organic anion analysis

Internal standard (40 μl of 100 μM ribitol) was added to the root exudates (10 ml aliquot), and lyophilized. Organic anions in exudates were analyzed according to Dias *et al*. [58]. Briefly, dried exudate samples were derivatized at 37°C in 20 μl of methoxyamine hydrochloride (30 mg/ml of in pyridine) (120 min), followed by 20 μl of *N*,*O*-bis(trimethylsilyl)trifluoroacetamide (BSTFA) with 1% trimethylchlorosilane (TMCS) (30 min). Samples (1 μl) were injected in an Agilent Gas Chromatograph 7890B coupled with a 7010 QqQ Mass Spectrometer (Agilent). Citrate, fumarate, malate, oxalate and succinate were identified and quantified using Agilent MassHunter software, version B.07.00.

### Statistical Analyses

Rarefaction analysis was performed with Analytic Rarefaction 1.3 [59]. Palaeontological Statistics (PAST) package ver. 3.07 [60] was used to calculate the Shannon diversity index, and differences in microbial community structures were analyzed with non-metric multidimensional scaling (NMDS) and one-way permutational multivariate analysis of variance (PERMANOVA) [61] with Bray-Curtis distance. Venn diagrams were generated with BioVenn [62]. Microbial taxa and functional genes associated with root types and the rhizosphere were elucidated with ANOVA using STAMP (STatistical Analysis of Metagenomic Profiles) ver. 2.1.3 [63, 64].

## Results

### Attachment to roots affects microbial communities in the rhizosphere

**Fig 1:**
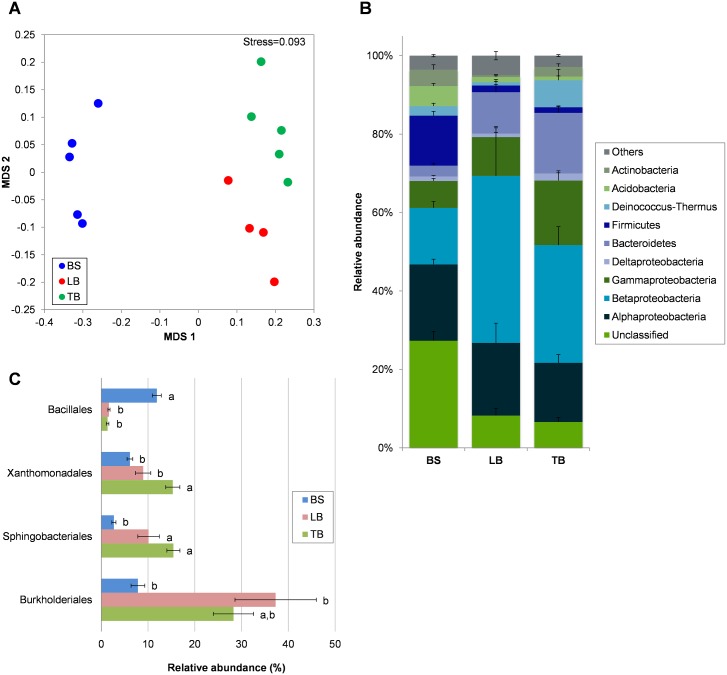
Bacterial community in the bulk soil (BS) and in the loosely-bound (LB) and tightly-bound (TB) fractions of the Brachypodium Bd21-3 rhizosphere, revealed with 16S pyrosequencing. (A) NMDS ordination plot (based on Bray-Curtis similarity), where each point represents the bacterial community in a soil/rhizosphere fraction for one plant. (B) Abundance of bacterial phyla in the bulk soil and rhizosphere (Proteobacteria is further classified into classes). (C) Bacterial Orders that are significantly different in abundance between the sample groups (different lower case letters indicate ANOVA *P*<0.05). Only Orders with >10% relative abundance in any sample type are shown. Means are shown ± SE (n = 4–5).

Bacterial and fungal communities of rhizosphere fractions were analyzed by next-generation sequencing (Experiment 1). A total of 1937 bacterial OTUs were identified from the 16S pyrosequencing, and only 131 OTUs were found overlapping between the bulk soil, loosely-bound and tightly-bound rhizospheres, while the majority were found to be unique to each sample type (S1 Table, S3 Fig). The mean numbers of OTUs identified in each sample were: 316, 213 and 201, for the bulk soil, loosely-bound, and tightly-bound rhizosphere fractions, respectively (S1 Table, S4 Fig). Shannon diversity indices of bacterial communities of the bulk soil, loosely-bound and tightly-bound rhizosphere fractions were 5.14, 3.99 and 4.01, respectively, with diversity in bulk soil significantly higher than loosely-bound and tightly-bound rhizospheres (ANOVA *P* = 0.0007 and 0.0011, respectively). This can also be confirmed from the rarefaction curves (S4 Fig). NMDS coupled with one-way PERMANOVA (Bray-Curtis distance) showed that bacterial community structures in bulk soil, loosely-bound and tightly-bound rhizospheres were significantly different from each other (*F* = 3.718, *P* = 0.0002), indicating that the communities were influenced by the distance from the root and how strongly attached they are (Fig 1A).

The class Betaproteobacteria and phylum Bacteroidetes were enriched in the loosely-bound and tightly-bound fractions, while Firmicutes, Acidobacteria and Actinobacteria were more prevalent in the bulk soil (Fig 1B). At the lower taxonomic levels, the bulk soil was dominated by the order Bacillales (phylum Firmicutes) which comprised 11.9% of the total bacterial population (Fig 1C). By contrast, the rhizosphere soil was dominated by the order Burkholderiales. This group constituted 37.3% of the loosely-bound fraction and 28.3% of the tightly-bound fractions but only 7.8% of the bulk soil (Fig 1C). The bacterial family most enriched in the rhizosphere was the Oxalobacteraceae (Burkholderiales) constituting 29.7% of the total population in the loosely-bound and 13.9% in the tightly-bound fraction (S1 Table). Order Sphingobacteriales was also enriched in the rhizosphere (loosely-bound and tightly-bound), and order Xanthomonadales was specifically enriched in the tightly-bound rhizosphere (Fig 1C).

**Fig 2:**
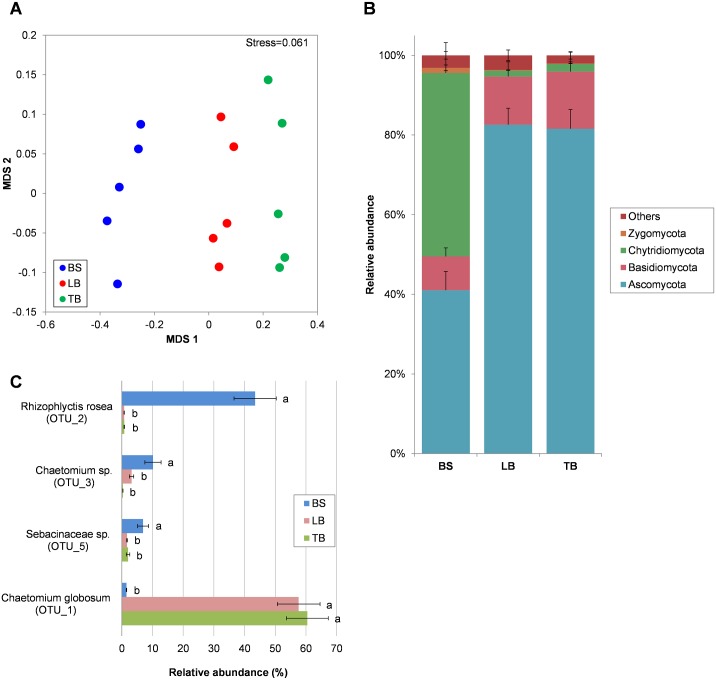
Fungal community in the bulk soil (BS) and in the loosely-bound (LB) and tightly-bound (TB) fractions of the Brachypodium Bd21-3 rhizosphere, revealed with ITS Illumina sequencing. (A) NMDS ordination plot (based on Bray-Curtis similarity, data were 4^th^ root transformed) where each point represents the fungal community in a soil/rhizosphere fraction for one plant. (B) Abundance of fungal phyla in the bulk soil and rhizosphere. (C) Fungal OTUs that are significantly different in abundance between the sample groups (different lower case letters indicate ANOVA *P*<0.05). Only OTUs with >5% relative abundance in any sample type are shown. Means are shown ± SE (n = 5).

Fungal communities in the rhizosphere were characterized by ITS Illumina sequencing, and a total of 554 OTUs were identified. Unlike the bacterial communities, more than half of the fungal OTUs (284 OTUs) overlapped with the bulk soil, loosely-bound and tightly-bound rhizospheres (S2 Table, S5 Fig). The mean numbers of OTUs identified in each sample were 321, 260 and 221 for the bulk soil, loosely-bound and tightly-bound rhizosphere fractions, respectively (rarefaction curves, S6 Fig), and no significant difference was observed in the fungal OTU diversity between bulk soil, loosely-bound and tightly-bound rhizospheres (Shannon diversity indices of 2.62, 1.94 and 1.79 respectively, ANOVA *P*>0.05). A single OTU comprised between 37 and 82% of sequences in all but one sample (S2 Table). This highly skewed distribution in all fractions explains the lack of a significant difference in the Shannon indices. The dominant fungal groups in loosely-bound and tightly-bound fractions were more similar to each other than to the bulk soil community (Figs 2B and 2C). In bulk soil the most abundant phyla were Chytridiomycota and Ascomycota, which comprised 46% and 41% of the total abundance, respectively (Fig 2B). Greater than 99% of Chytridiomycota sequences were classified as *Rhizophlyctis rosea* and this OTU dominated the bulk soil (Fig 2C). In the loosely-bound and tightly-bound rhizosphere fractions the Ascomycota was enriched to 82–83% of the total population while the abundance of Chytridiomycota dropped to 1.5–1.9% (Fig 2B). The enrichment of Ascomycota in the rhizosphere was largely driven by the most abundant OTU, classified as *Chaetomium globosum* (Order Sordariales), which increased 37 to 49-fold compared to the bulk soil (Fig 2C). Despite apparent similarities in the fungal composition in the rhizosphere fractions (Figs 2B and 2C) the communities were significantly different in bulk soil, loosely-bound and tightly-bound samples (PERMANOVA *F* = 6.245, *P* = 0.0001) when sequencing data were 4^th^ root transformed prior to calculating distances (Fig 2A). This indicates that even though the dominant groups (OTU) were similar between the loosely-bound and tightly-bound rhizosphere fractions, the minor components were significantly different. For example, *Emericellopsis mirabilis* (Ascomycota) showed greater enrichment in the tightly-bound rhizosphere (~1% of sequences) compared to the loosely-bound rhizosphere (~0.1% of sequences, ANOVA *P* = 0.001) (S2 Table).

### Different root types harbour different microbial communities

The bacterial and fungal communities colonizing the seminal and nodal roots of Brachypodium were sampled 30 and 44 DAS (days after sowing) (Experiment 2) and analyzed with 16S and ITS T-RFLP. Firstly microbial communities on the different root types were significantly different for bacteria (PERMANOVA *F* = 3.199, *P* = 0.0001) and fungi (PERMANOVA *F* = 1.764, *P* = 0.0001) but the bacterial communities showed the largest changes (S7 Fig). These differences in community structure were not related to incubation period or to depth of the root in the pot because bacterial and fungal communities sampled from the top, middle and bottom layers of the bulk soil showed no significant differences (PERMANOVA *F* = 1.015, *P* = 0.433 for bacteria, and *F* = 1.051, *P* = 0.299 for fungi) (S8 Fig).

**Fig 3:**
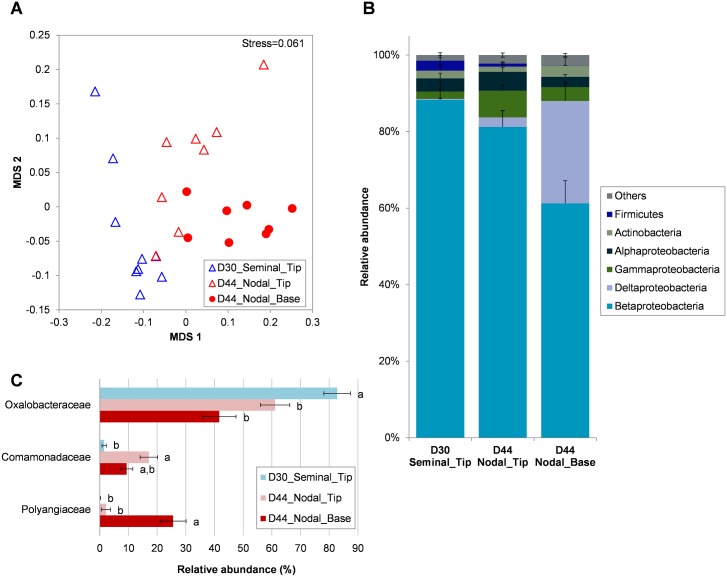
16S pyrosequencing revealed the bacterial communities colonizing the seminal root tip at day 30, and the root tip and base of nodal roots at day 44. (A) NMDS ordination plot (based on Bray-Curtis similarity) where each point represents the bacterial community in one root sample. (B) Abundance of bacterial phyla in each root type (Proteobacteria further classified into classes). (C) Bacterial families significantly different in abundance between root types (different lower case letters indicate ANOVA *P*<0.05). Only Orders with >10% relative abundance in any root type are shown. Data are means ± SE (n = 8–9).

We further analyzed the bacterial communities colonizing the seminal and nodal roots with 16S pyrosequencing. The samples analyzed included seminal root tips at 30 DAS (D30_Seminal_Tip) and nodal root tips and bases at 44 DAS (D44_Nodal_Tip and D44_Nodal_Base respectively). These samples were chosen because the seminal roots at 30 DAS were the same age as nodal roots 44 DAS and because they showed distinct T-RFLP profiles (S7A Fig). Since roots were washed twice before sampling it is likely the communities analyzed here were the endorhizosphere and the rhizoplane populations, which are firmly attached on the root surface. A total of 1800 OTUs were identified from the sequencing run, and the majority of the OTUs were found to be unique to each sample type (S3 Table, S9 Fig). The mean OTU numbers identified in each sample were 120, 155 and 167 for D30_Seminal_Tip, D44_Nodal_Tip and D44_Nodal_Base respectively. Shannon diversity indices for D30_Seminal_Tip, D44_Nodal_Tip and D44_Nodal_Base were 1.39, 2.07 and 2.26 respectively, and the bacterial diversity for D44_Nodal_Base was significantly greater than D30_Seminal_Tip (ANOVA *P* = 0.04) (rarefaction curves, S10 Fig). OTU-based NMDS showed that community structures can be grouped according to the sample type, and they were significantly different between D30_Seminal_Tip, D44_Nodal_Tip and D44_Nodal_Base (PERMANOVA *F* = 7.459, *P* = 0.0001) (Fig 3A). More than 97% of the OTUs can be classified into six major bacterial phyla (Betaproteobacteria, Deltaproteobacteria, Gammaproteobacteria, Alphaproteobacteria, Actinobacteria and Firmicutes) with the most abundant, Betaproteobacteria, comprising 88.3, 81.2 and 61.2% of the samples in D30_Seminal_Tip, D44_Nodal_Tip and D44_Nodal_Base, respectively (S3 table, Fig 3B). The taxonomic composition of the bacterial communities in the seminal and nodal roots was much simpler compared to the bulk soil or the rhizosphere samples in Experiment 1 (Fig 1B) because a single family, Oxalobacteraceae (Betaproteobacteria), dominated the community with 82.8, 61.1 and 41.7% of the population in D30_Seminal_Tip, D44_Nodal_Tip and D44_Nodal_Base, respectively (Fig 3C). Family Commamonadaceae (Betaproteobacteria) was strongly associated with nodal roots as the population was more abundant in the D44_Nodal_Tip and D44_Nodal_Base compared to the D30_Seminal_Tip (ANOVA *P* = 0.0003 and 0.054 compared to the D30_Seminal_Tip, respectively). Deltaproteobacteria (predominantly family Polyangiaceae) only contributed 0.2% and 2.5% of the population in D30_Seminal_Tip and D44_Nodal_Tip respectively, but was significantly enriched to 26.7% in the D44_Nodal_Base, indicating that this group is more strongly associated with the root base (Figs 3B and 3C). Gammaproteobacteria were found to be relatively more abundant in D44_Nodal_Tip (6.9%), compared to D30_Seminal_Tip (1.9%) and D44_Nodal_Base (3.6%) (Fig 3B).

### Predicted gene functions reveal differences in bacterial function between bulk and rhizosphere soils and between root types

**Fig 4:**
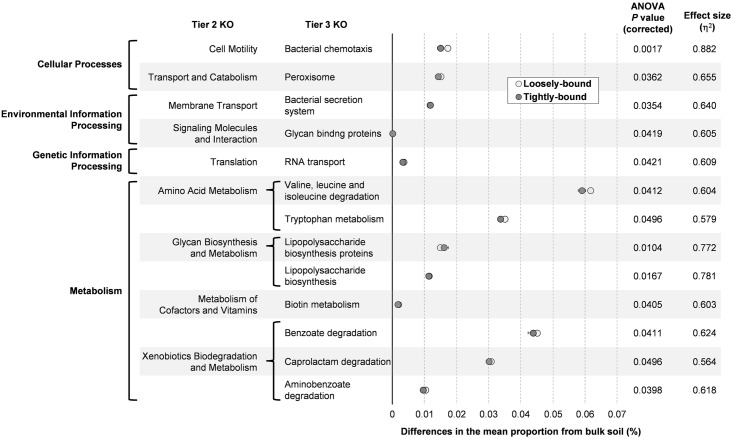
PICRUSt predicted bacterial functional gene content that was increased in the Brachypodium rhizosphere. Differences in the abundance of categorized gene functions (tier 3 KO) in the loosely-bound and tightly-bound rhizospheres are plotted against the bulk soil (= 0 on the x-axis). Only categories that were significantly more abundant in the rhizospheres (both loosely-bound and tightly-bound) compared to bulk soil are shown (ANOVA, Benjamini-Hochberg FDR corrected *P* < 0.05). Data are means ± SE (n = 4–5).

**Fig 5:**
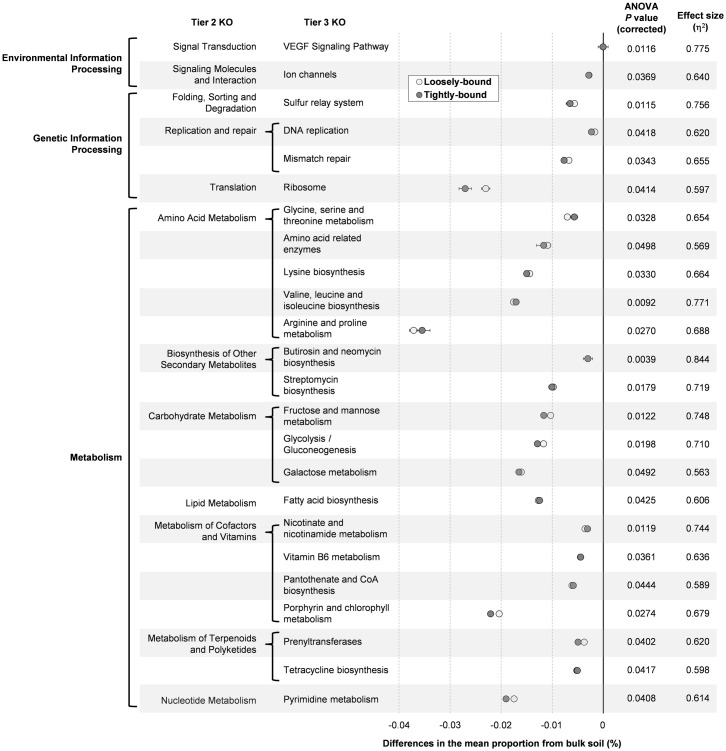
PICRUSt predicted bacterial functional gene content that was decreased in the Brachypodium rhizosphere. Differences in the abundance of categorized gene functions (tier 3 KO) in the loosely-bound and tightly-bound rhizospheres are plotted against the bulk soil (= 0 on the x-axis). Only categories that were significantly decreased in the rhizospheres (both loosely-bound and tightly-bound) compared to bulk soil are shown (ANOVA, Benjamini-Hochberg FDR corrected *P* < 0.05). Data are means ± SE (n = 4–5).

To assess the functional capabilities of bacterial communities colonizing Brachypodium rhizosphere fractions, the bacterial metagenome was predicted from the 16S amplicon data using PICRUSt. The predicted metagenome was compared at the tier 3 KEGG Orthology (KO) for the bulk soil and the rhizosphere bacterial communities (loosely-bound and tightly-bound to root surfaces). There were 13 functional gene categories at tier 3 KO that were significantly more abundant in the rhizosphere than the bulk soil (ANOVA, Benjamini-Hochberg FDR corrected *P*<0.05) (Fig 4). Eight ‘metabolism’ pathways were enhanced in the loosely-bound and tightly-bound rhizospheres. Two of these are involved with lipopolysaccharide biosynthesis, a major component of outer membrane of Gram-negative bacteria (Fig 4). This change corresponds to the reduction of Gram-positive bacteria (Firmicutes) in the rhizosphere (Fig 1B). Functional genes involved in ‘bacterial chemotaxis’ were also more abundant in the rhizosphere (Fig 4). On the other hand, there were 24 functional gene categories at tier 3 KO that were significantly decreased in the rhizosphere (both loosely-bound and tightly-bound) (ANOVA, Benjamini-Hochberg FDR corrected *P*<0.05) (Fig 5). The majority of the decreased functional gene categories (18 gene categories) are involved in the ‘metabolism’ pathways, including genes involved in the metabolism of various amino acids, carbohydrates, cofactors and vitamins. No gene categories involved in the ‘cellular processes’ pathways were decreased in the rhizosphere (Fig 5).

**Fig 6:**
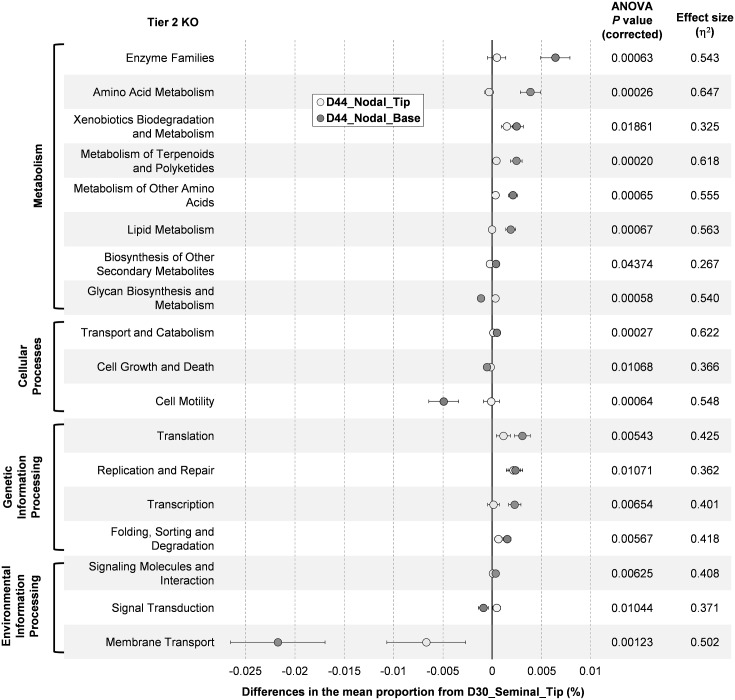
Bacterial functional gene content in the Brachypodium seminal and nodal roots inferred by PICRUSt. Differences in the abundance of categorized gene functions (tier 2 KO) between the nodal roots (D44_Nodal_Tip and D44_Nodal_Base) are plotted against seminal roots (D30_Seminal_Tip) (= 0 on the x-axis). Only categories that were significantly different in abundance are shown (ANOVA, Benjamini-Hochberg FDR corrected *P* < 0.05). Data are means ± SE (n = 8–9).

The inferred metagenome of bacterial populations colonizing seminal and nodal roots was compared at the tier 2 KO. The abundance of 18 functional gene categories was found to be significantly different between D30_Seminal_Tip, D44_Nodal_Tip and D44_Nodal_Base (ANOVA, Benjamini-Hochberg FDR corrected *P*<0.05) (Fig 6). Despite differences in bacterial populations between the D30_Seminal_Tip and the D44_Nodal_Tip (see Fig 3A), the functional gene contents on these was very similar, and quite distinct from those on the basal root tissue (D44_Nodal_Base; Fig 6). Seven gene categories with ‘metabolism’-related functions were significantly enhanced in the D44_Nodal_Base, while genes involved in ‘cell motility’ were more abundant on root tips of seminal and nodal roots. Genes categorized as having ‘genetic information processing’-related functions appeared to be more enhanced in the nodal roots (tip and base) than the seminal root, and genes involved in ‘membrane transport’ were more abundant in the seminal root tips than the nodal roots (tip and base) (Fig 6).

### Root exudation of Brachypodium

**Fig 7:**
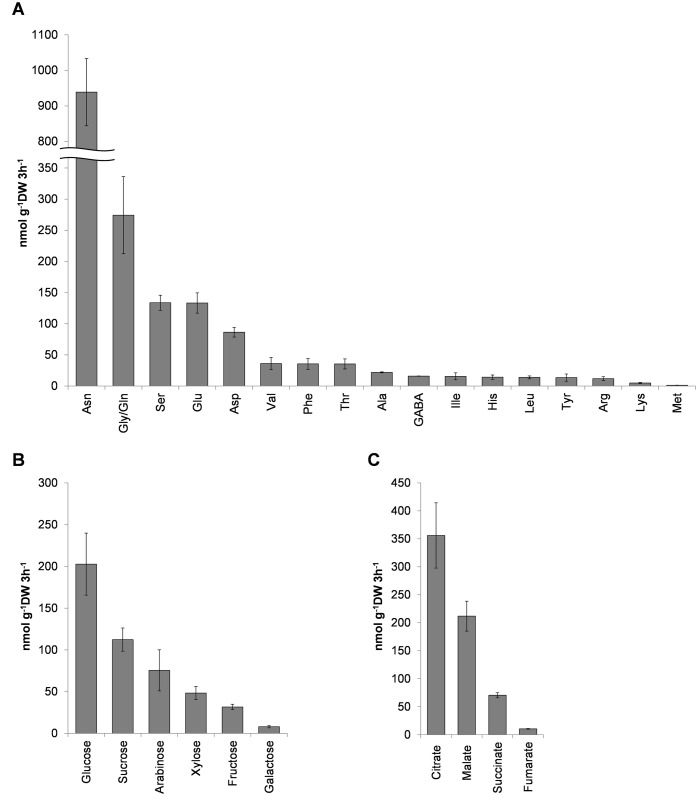
Root exudates composition of Brachypodium. Amount of (A) amino acids, (B) sugars, and (C) organic anions released from Brachypodium roots in 3h root exudate collection period. Data are means ± SE (n = 4 (A and B) or 9 (C)).

Root exudates have a major impact on the composition of the bacterial and fungal communities in the rhizosphere. Therefore the major components of Brachypodium root exudates (amino acids, sugars and organic acids) were characterized and quantified with HPLC and GC-MS. Eighteen amino acids were released from Brachypodium roots, and asparagine was the most abundant with 940 nmol g^-1^DW 3h^-1^ (Fig 7A). Serine, glutamic and aspartic acids were the next most abundant at 86.4–133.8 nmol g^-1^DW 3h^-1^, while the least abundant amino acid released was methionine with 1.3 nmol g^-1^DW 3h^-1^. Glycine and glutamine could not be separated with the HPLC and cysteine, proline and tryptophan were not detected (Fig 7A). The six sugars released from roots were glucose, sucrose, arabinose, xylose, fructose and galactose. Glucose was the most abundant and galactose the least abundant showing ~203 and 8.0 nmol g^-1^DW 3h^-1^, respectively (Fig 7B). The most abundant organic anion in the root exudates was citrate (356.0 nmol g^-1^DW 3h^-1^), followed by malate (212 nmol g^-1^DW 3h^-1^), succinate (70 nmol g^-1^DW 3h^-1^) and fumarate (10 nmol g^-1^DW 3h^-1^) (Fig 7C). Oxalate was detected but could not be quantified since its peak co-eluted with other compounds.

## Discussion

The aim of the present study was to provide details of the Brachypodium rhizosphere for use as a model plant for research in temperate cereals, likely wheat. Watt *et al*. [15] characterized the root architecture of Brachypodium including anatomical differences among nodal and seminal roots, and their similarity to those of wheat. In this study we described the microbial communities on and near Brachypodium roots in detail, measured spatial variation according to root types and along roots, and quantified the exudate profiles of sugars, amino acids and organic anions, providing foundational information for the use of Brachypodium as a model temperate cereal.

Brachypodium bacterial and fungal communities were significantly different among bulk soil, the loosely-bound rhizosphere and the tightly-bound rhizosphere (Figs 1A and 2A). Distance and adhesion to the roots influenced microbial composition. Corgié *et al*. [65] found that the number of culturable bacteria decreased, and community structure changed, over the 3 mm distance away from root surfaces. Donn *et al*. [31] reported distinct bacterial communities in loosely-bound and tightly-bound rhizosphere fractions on field-grown wheat. These differences are likely to be influenced, in part, by the gradient in compounds released by roots. Indeed, ^14^C labelling revealed that 80% of released carbon remained within 2 mm from the root surface, although exudates from some species diffused as far as 10 mm from root surfaces [66]. Properties of the root surface biofilm that retains specific bacteria also likely influence differences in tightly and loosely bound rhizosphere communities [67].

The composition of bacteria in the rhizosphere was similar for Brachypodium and wheat despite plants being grown in different soils and environments. Donn *et al*. [31] examined microorganisms in loosely and tightly-bound rhizospheres on field-grown wheat roots and showed a low abundance of Firmicutes, and enrichment of Bacteroidetes and Betaproteobacteria. The tightly-bound rhizosphere was also specifically enriched in Gammaproteobacteria [31]. These changes reflect the general pattern reported here for Brachypodium (Fig 1B) and illustrate the similar influence these species have on the bacterial populations around their roots.

One difference between wheat and Brachypodium were Actinobacteria, which were highly abundant in the rhizosphere of field-grown wheat [31, 32], but not Brachypodium. Actinobacteria consume organic matter in soil and the richness of Actinobacteria depends on the quality and quantity present [68]. In the present study plants were grown in a mixture of sand (80%) and soil (20%). The low organic matter may not have been conducive to the proliferation of Actinobacteria. This is consistent with findings of Tkacz *et al*. [35] who found fewer Actinobacteria in rhizospheres of Brachypodium and other species grown in a sand (90%) and soil (10%) medium than in a compost (90%) and soil (10%) mixture [35]. Enrichment of root-associated Actinobacteria appears to depend on the organic matter content of the soil and plant-related traits.

At lower taxonomic levels, the order Burkholderiales (phylum Proteobacteria) was enriched in the rhizosphere of Brachypodium and represented approximately 30% of the loosely-bound and tightly-bound communities (Fig 1C). Within the Burkholderiales, the Oxalobacteraceae family was most abundant (S1 Table). A similar enrichment was reported in wheat [31, 32]. By contrast, in the model eudicot species, Arabidopsis, enrichment of the Burkholderiales (Oxalobacteraceae) in the rhizosphere was not observed [30]. Members of Oxalobacteraceae includes a well-known plant growth-promoting bacterium *Herbaspirillum seropedicae*, and this species is known to endophytically colonize Brachypodium roots and enhances growth of some Brachypodium genotypes under N stress [11]. Interestingly, some members of Oxalobacteraceae utilize oxalate as a carbon source [69] and we also detected oxalate in the Brachypodium root exudates.

Fungal populations in the bulk soil and rhizosphere of Brachypodium were dominated by the Order Sordariales (phylum Ascomycota). An OTU classified as *Chaetomium globosum* accounted for more than 50% of all sequences (Fig 2C). This species can have plant growth promoting effects through phosphorus mobilization [70] or disease suppression [71–73]. The most abundant fungal species in the bulk soil, *Rhizophlyctis rosea* (Chytridiomycota), was a saprotrophic cellulose decomposer [74]. This was largely excluded from the rhizosphere (Fig 2C). High abundance of the Chytridiomycota in the bulk soil community (up to 63%) is common in Australian soils, especially for disturbed soils [75].

Microbial communities are also known to vary along the length of a root [76–78], with root age [79, 80], and root types (e.g. between seminal root system and nodal root system) [81]. However, the majority of rhizosphere microbiome studies have not considered root type, despite reported differences between seed- and shoot-borne roots in function and development [13], as well as in their structural [82] and biophysical [83] properties. Brachypodium is known to develop three types of axile roots. A primary seminal root emerges from the base of the embryo at germination. Then up to two coleoptile nodal roots emerge above the seed at leaf 3 stage, and then multiple leaf nodal roots emerge from stem nodes associated with the leaves by leaf 5 [14, 15]. These axile roots are different in their vascular anatomies, especially between the seminal root and the nodal roots (both coleoptile and leaf nodal roots) [15]. Brachypodium line Bd21-3,used in this study, is known to develop a relatively small coleoptile nodal root system [13], and in our system, most of the plants did not develop coleoptile nodal roots. Therefore, we only analyzed the microbial population on the seminal and the leaf nodal root systems. We sampled 4 cm sections of the tips (younger sections) and bases (older sections) of each axile root type and sequencing revealed that bacterial and fungal communities in the rhizospheres were significantly influenced by root age, root type, and by position along a root (tip or base) (Fig 3A and S7 Fig). Oxalobacteraceae were enriched strongly on seminal and nodal roots, while Comamonadaceae was only enriched on the tip and base of nodal roots (Fig 3C). The family Polyangiaceae (class Deltaproteobacteria) was specifically enriched at the base of nodal roots (Fig 3C), and the genus *Sorangium* was the most abundant. This genus can degrade cellulose readily and is often isolated from decaying plant materials [84]. Enrichment of *Sorangium* at the root base may correlate with the decaying of cortical cells that are older than those at the tips. *Sorangium* produces fungicides and bactericides [85], and members may serve as biocontrol agents in the rhizosphere. These results demonstrate that averaging the microbial community structure across an entire root system will obscure important variation in members of the microbiome associated with specific root types and locations.

Gene content of populations was used to identify differences in bacterial functions between the bulk soil and rhizosphere (Figs 4 and 5). Some categories of gene function were clearly more abundant in the rhizosphere (Fig 4), including the ‘bacterial chemotaxis’ function to detect and respond to physicochemical gradients common in the rhizosphere, perhaps favouring the movement of bacteria toward root exudates [86, 87]. Another functional group enriched in the rhizosphere was the ‘bacterial secretion system’. These allow bacteria to interact with their environment, more readily forming mutualistic or pathogenic associations with host plants [88]. Rhizosphere enrichment of bacterial secretion systems is consistent with previous findings in soybean [89]. ‘Xenobiotics biodegradation’ functions were also more abundant in the rhizosphere than bulk soil. Plants, possibly including Brachypodium, produce a wide range of secondary metabolites many of which are structurally similar to xenobiotics [90]. On the other hand, the majority of gene categories that appeared to be more abundant in the bulk soil compared to the rhizosphere (i.e. decreased in the rhizosphere) are related to ‘metabolism’ pathways (Fig 5). This may be explained by the fact that plant litter materials (e.g. polysaccharides and lignin) are the major organic matters in soil [91], and the bulk soil microbes needs a more complex enzyme system to utilize these macro molecules [92].

Location along a root (tip or base) had a larger influence on bacterial functions than root type. Bacterial functions on tips of nodal roots were more similar to tips of seminal roots than to the base of nodal roots (Fig 6). Age of tissue (e.g. tips with dividing and elongating cells) may determine associated bacterial functions more than root type. Gene function categories denoted as ‘cell motility’ and ‘membrane transport’ were more abundant at root tips (Fig 6). The ‘membrane transport’ category (e.g. transporter proteins) enable bacteria to interact with the surroundings [88] and motility facilitates chemotactic responses to chemical gradients generated by root exudates and other signals in the rhizosphere [93]. Similarly, genes for metabolic processes were more abundant in the base of roots than the root tips. Complex enzyme systems are required to degrade macromolecules such as cellulose and lignin [92], and bacteria at root bases may be associated with the cortical cell senescence that occurs on root aging.

Rhizodeposits including the root exudates are the major carbon sources for the rhizosphere microorganisms, and the components of root exudates determine the composition of the root colonizing microbes [20]. Therefore, it is important to catalogue the exudate content of the plant, in order to have a better understanding of the root-microbe interactions in the rhizosphere of specific plant species. Amino acids, sugars and organic anions are major components of root exudates of plants, and can influence or be influenced by the structure of the microbial community [94, 95]. Amino acids are the largest class of released nitrogenous compounds in wheat [96]. We detected 18 amino acids in the exudates of Brachypodium roots, with asparagine released in largest quantities followed by serine and glutamic acid (Fig 7A). Glutamic acid and serine are also common to wheat and maize exudates, but alanine can be most abundant [94, 96–98]. Brachypodium therefore appears to share common amino acid exudates to wheat, but differences may depend on species or environments used for collection.

Sugars can constitute ~70% of total root exudate carbon [99]. Six sugars were identified in Brachypodium exudates: glucose, sucrose, arabinose, xylose, fructose and galactose. All have been identified in exudates of Arabidopsis [100], rice [93], maize [98], wheat and barley [101]. The composition of sugars released from Brachypodium resembled that of wheat with glucose being the most abundant and galactose the least abundant (Fig 7B) [101]. Malate, citrate, succinate, fumarate and oxalate are the organic acids most commonly released from plants [98, 102, 103], and these were also released from Brachypodium roots. Citrate was the most abundant followed by malate, succinate and fumarate (Fig 7C).

In summary, Brachypodium was found to be a suitable model for the rhizosphere of wheat due to their strong resemblance in both microbial populations and root exudates. Brachypodium accessions show variation in their dry matter allocations to nodal and seminal roots which, in turn, reflect different responses to water availability [13]. Therefore it is possible that the variation in phylogeny and function of microbial populations on different root types, demonstrated here, could be exploited to select microbiomes with contrasting functions.

## Supporting Information

S1 Fig A scanned image of a *B*. *distachyon* Bd21-3 root system 30 days after sowing. The axile root of the seminal root system is indicated by a yellow broken line. Tips and bases of the nodal root and the seminal root were sampled (4 cm each), and these includes the lateral roots branched from the axile root.
(PDF)

S2 Fig Brachypodium root exudates collection system. (A) Brachypodium sterile semi-hydroponic system. Plants were grown in a tissue culture container, filled with 2 mm glass beads and saturated with ¼ Hoagland’s solution. (B) Root exudate collection. Plants were carefully removed from the semi-hydroponic system, the roots were rinsed well and immersed in 20 ml of ultra-pure water in a glass jar. The root exudates were collected for 3 hours.
(PDF)

S3 Fig Venn diagram showing the number of shared and unique bacterial OTUs identified in bulk soil and Brachypodium rhizospheres. An OTU was considered unique if it was present in at least one replicate of one group, and absent in the other groups (n = 4–5).
(PDF)

S4 Fig Rarefaction curves of bacterial OTUs identified in the bulk soil and Brachypodium rhizospheres. Bacterial community was analyzed with 16S pyrosequencing, and 1015 sequences were randomly subsampled from each sample to achieve even sequencing depth. Means are shown ± SE (n = 4–5).
(PDF)

S5 Fig Venn diagram showing the number of shared and uniqe fungal OTUs identified in bulk soil and Brachypodium rhizospheres. An OTU was considered unique if it was present in at least one replicate of one group, and absent in the other groups (n = 5).
(PDF)

S6 Fig Rarefaction curves of fungal OTUs identified in the bulk soil and Brachypodium rhizospheres. Fungal community was analyzed with ITS Illumina sequencing, and 49096 sequences were randomly subsampled from each sample to achieve even sequencing depth. Means are shown ± SE (n = 5).
(PDF)

S7 Fig Microbial community structures of Brachypodium roots. NMDS ordination plots (Bray-Curtis similarity) of (A) bacterial and (B) fungal community structures colonizing Brachypodium seminal and nodal root tips and bases at day 30 and day 44 after sowing. Bacterial 16S rRNA and fungal ITS gene diversities were analyzed with T-RFLP. Data are mean NMDS scores for axes 1 and 2 ± SE (n = 9).
(PDF)

S8 Fig Microbial community structures in bulk soil. NMDS ordination plots (Bray-Curtis similarity) of (A) bacterial and (B) fungal community structures in the top, middle and bottom layer of the bulk soil samples (unvegetated pot) at day 30 and day 44 after incubation. Bacterial 16S rRNA and fungal ITS gene diversities were analyzed with T-RFLP. Data are mean NMDS scores for axes 1 and 2 ± SE (n = 4).
(PDF)

S9 Fig Venn diagram showing the number of shared and unique bacterial OTUs identified in Brachypodium seminal and nodal root systems. An OTU was considered unique if it was present in at least one replicate of one group, and absent in the other groups (n = 8–9).
(PDF)

S10 Fig Rarefaction curves of bacterial OTUs identified in Brachypodium seminal and nodal root systems. Bacterial community was analyzed with 16S pyrosequencing, and 3534 sequences were randomly subsampled from each sample to achieve even sequencing depth. Means are shown ± SE (n = 8–9).
(PDF)

S1 Table Bacterial OTU counts in the bulk soil and Brachypodium rhizospheres. Bacterial communities were analyzed with 16S pyrosequencing, and the taxonomy was assigned according to the RDP database.
(XLSX)

S2 Table Fungal OTU counts in the bulk soil and Brachypodium rhizospheres. Fungal communities were analyzed with ITS Illumina sequencing, and the taxonomy was assigned according to the UNITE ITS database.
(XLSX)

S3 Table Bacterial OTU counts in the seminal and nodal root systems of Brachypodium. Bacterial communities were analyzed with 16S pyrosequencing, and the taxonomy was assigned according to the RDP database.
(XLSX)
