# Supplementary material for: Microbiome and Exudates of the Root and Rhizosphere of Brachypodium distachyon, a Model for Wheat
Source: PLoS One. 2016 Oct 11;11(10):e0164533. doi: 10.1371/journal.pone.0164533 (PMC5058512; doi:10.1371/journal.pone.0164533)
Supplement: S1 Fig — The axile root of the seminal root system is indicated by a yellow broken line. Tips and bases of the nodal root and the seminal root were sampled (4 cm each), and these includes the lateral roots branched from the axile root. (PDF) [file pone.0164533.s001.pdf]

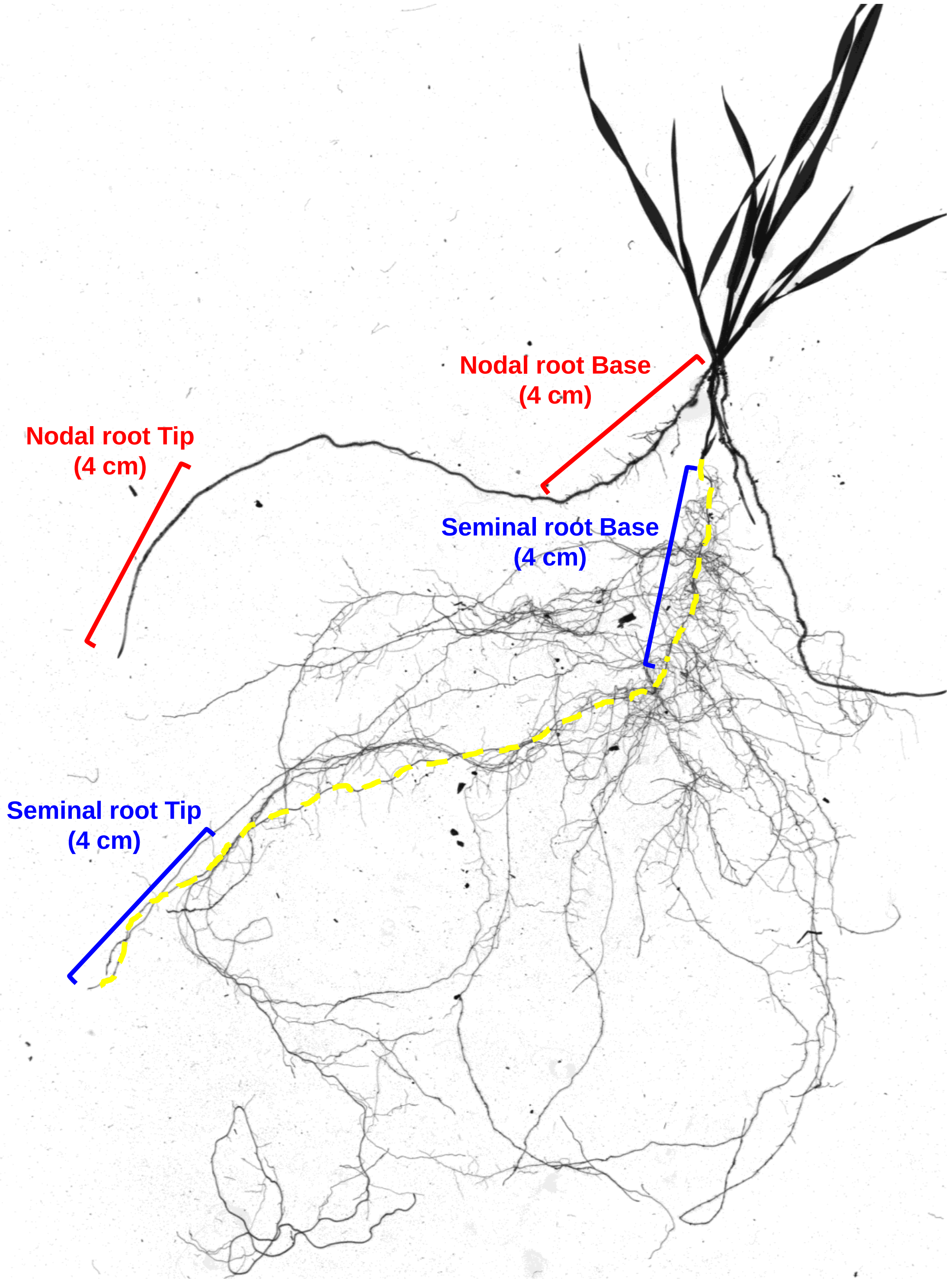

S1 Fig. A scanned image of a *B. distachyon* Bd21-3 root system 30 days after sowing. The axile root of the seminal root system is indicated by a yellow broken line. Tips and bases of the nodal root and the seminal root were sampled (4 cm each), and these includes the lateral roots branched from the axile root.
