# Supplementary material for: Microbiome and Exudates of the Root and Rhizosphere of Brachypodium distachyon, a Model for Wheat
Source: PLoS One. 2016 Oct 11;11(10):e0164533. doi: 10.1371/journal.pone.0164533 (PMC5058512; doi:10.1371/journal.pone.0164533)
Supplement: S2 Fig — (A) Brachypodium sterile semi-hydroponic system. Plants were grown in a tissue culture container, filled with 2 mm glass beads and saturated with ¼ Hoagland’s solution. (B) Root exudate collection. Plants were carefully removed from the semi-hydroponic system, the roots were rinsed well and immersed in 20 ml of ultra-pure water in a glass jar. The root exudates were collected for 3 hours. (PDF) [file pone.0164533.s002.pdf]

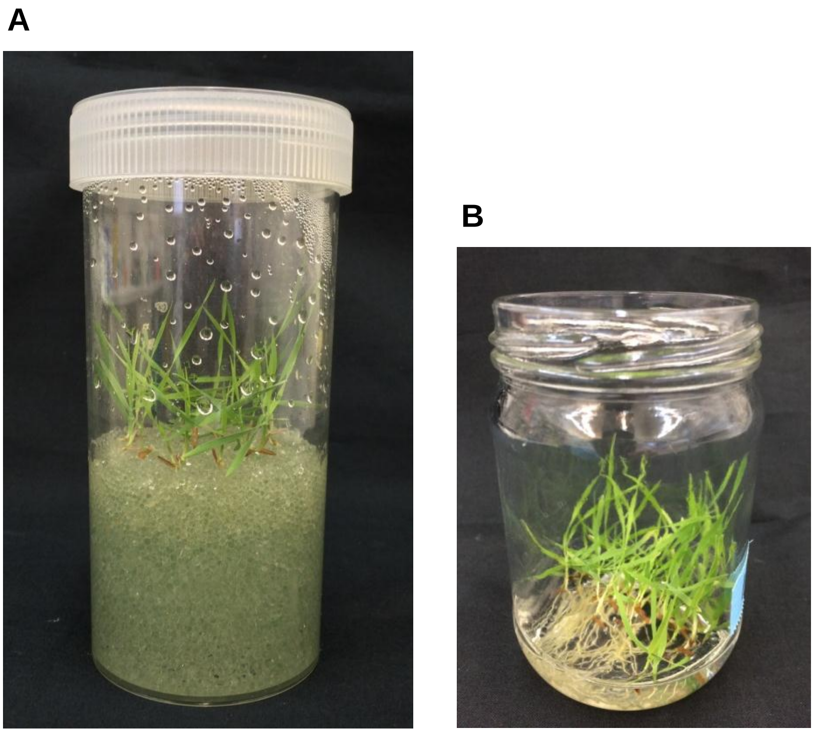

S2 Fig. Brachypodium root exudates collection system. (A) Brachypodium sterile semi-hydroponic system. Plants were grown in a tissue culture container, filled with 2 mm glass beads and saturated with ¼ Hoagland’s solution. (B) Root exudate collection. Plants were carefully removed from the semi-hydroponic system, the roots were rinsed well and immersed in 20 ml of ultra-pure water in a glass jar. The root exudates were collected for 3 hours.
