# Supplementary material for: Microbiome and Exudates of the Root and Rhizosphere of Brachypodium distachyon, a Model for Wheat
Source: PLoS One. 2016 Oct 11;11(10):e0164533. doi: 10.1371/journal.pone.0164533 (PMC5058512; doi:10.1371/journal.pone.0164533)
Supplement: S3 Fig — An OTU was considered unique if it was present in at least one replicate of one group, and absent in the other groups (n = 4–5). (PDF) [file pone.0164533.s003.pdf]

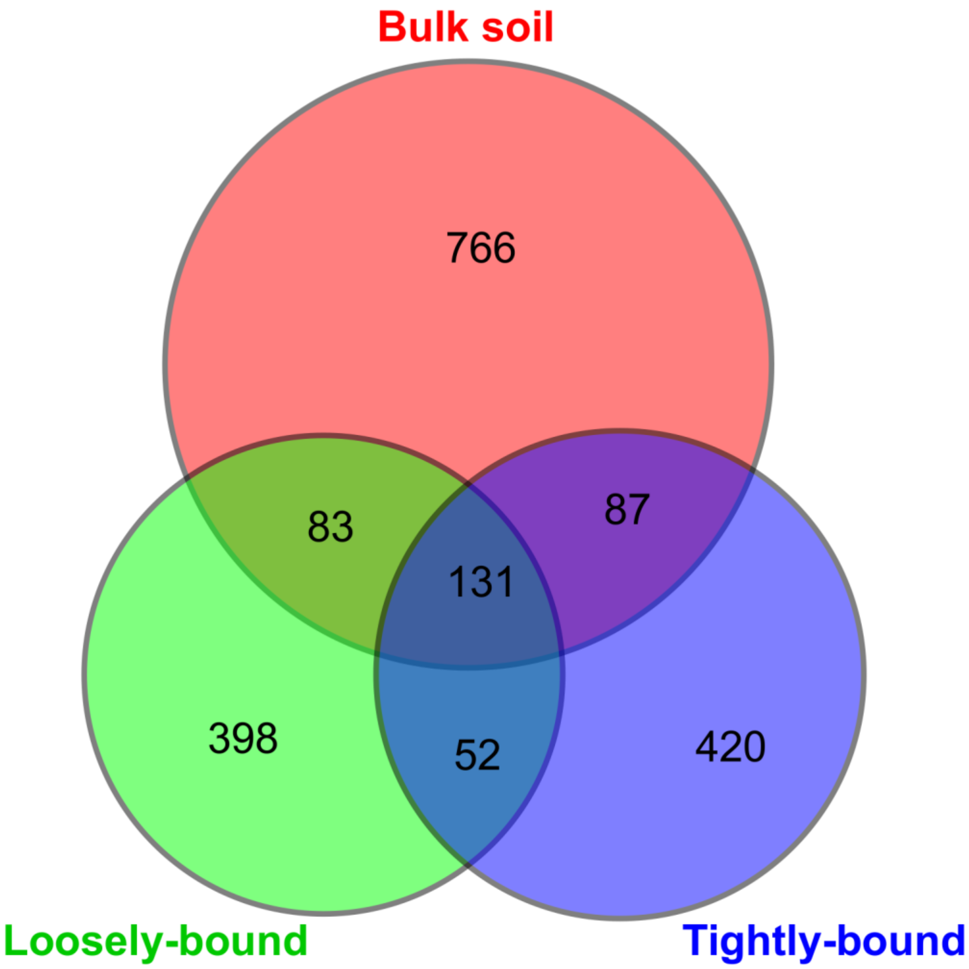

S3 Fig. Venn diagram showing the number of shared and unique bacterial OTUs identified in bulk soil and Brachypodium rhizospheres. An OTU was considered unique if it was present in at least one replicate of one group, and absent in the other groups (n=4-5).
