# Supplementary material for: Microbiome and Exudates of the Root and Rhizosphere of Brachypodium distachyon, a Model for Wheat
Source: PLoS One. 2016 Oct 11;11(10):e0164533. doi: 10.1371/journal.pone.0164533 (PMC5058512; doi:10.1371/journal.pone.0164533)
Supplement: S4 Fig — Bacterial community was analyzed with 16S pyrosequencing, and 1015 sequences were randomly subsampled from each sample to achieve even sequencing depth. Means are shown ± SE (n = 4–5). (PDF) [file pone.0164533.s004.pdf]

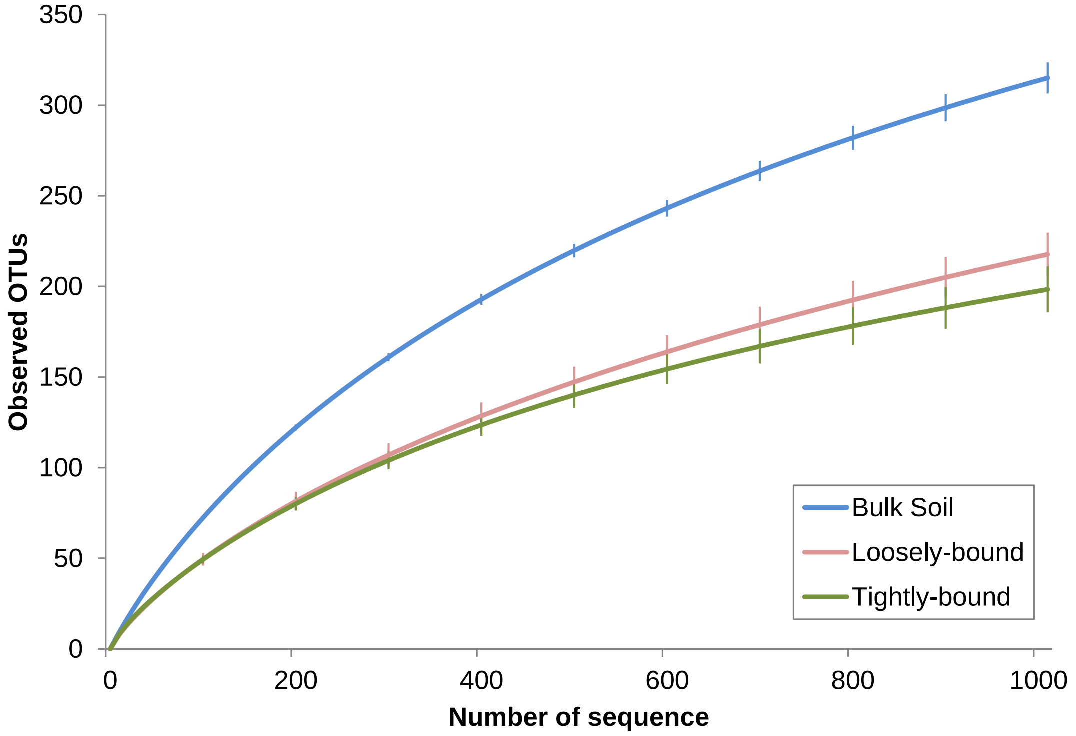

S4 Fig. Rarefaction curves of bacterial OTUs identified in the bulk soil and Brachypodium rhizospheres. Bacterial community was analyzed with 16S pyrosequencing, and 1015 sequences were randomly subsampled from each sample to achieve even sequencing depth. Means are shown ± SE (n=4-5).
