# Supplementary material for: Microbiome and Exudates of the Root and Rhizosphere of Brachypodium distachyon, a Model for Wheat
Source: PLoS One. 2016 Oct 11;11(10):e0164533. doi: 10.1371/journal.pone.0164533 (PMC5058512; doi:10.1371/journal.pone.0164533)
Supplement: S6 Fig — Fungal community was analyzed with ITS Illumina sequencing, and 49096 sequences were randomly subsampled from each sample to achieve even sequencing depth. Means are shown ± SE (n = 5). (PDF) [file pone.0164533.s006.pdf]

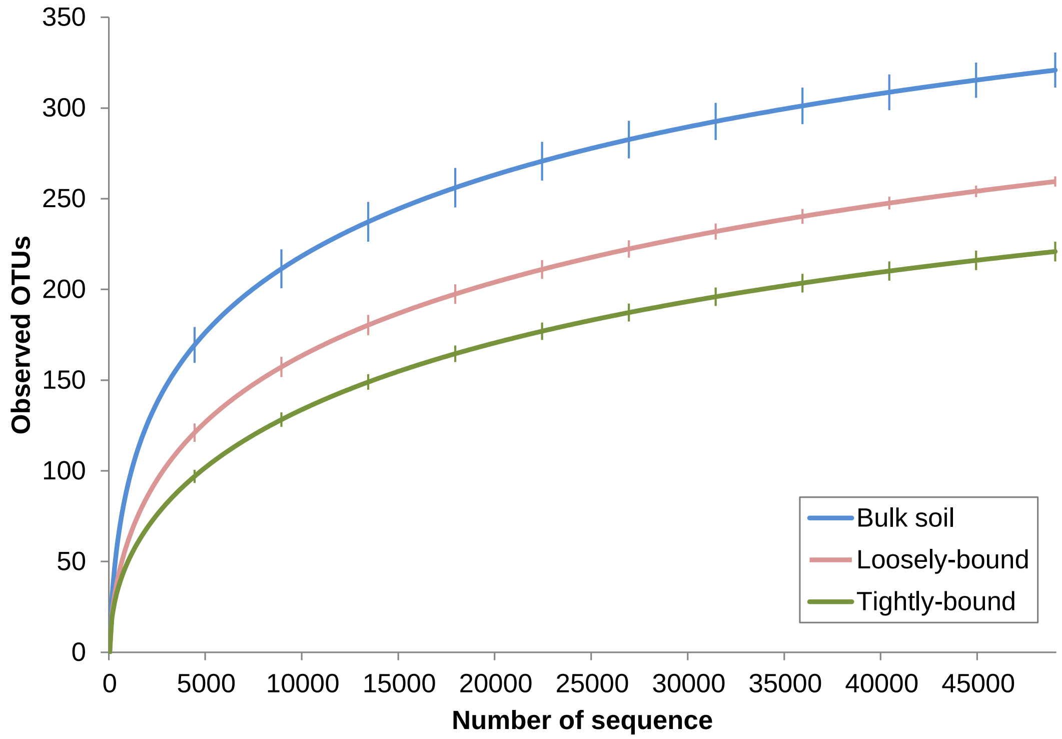

S6 Fig. Rarefaction curves of fungal OTUs identified in the bulk soil and Brachypodium rhizospheres. Fungal community was analyzed with ITS Illumina sequencing, and 49096 sequences were randomly subsampled from each sample to achieve even sequencing depth. Means are shown ± SE (n=5).
