# Supplementary material for: Microbiome and Exudates of the Root and Rhizosphere of Brachypodium distachyon, a Model for Wheat
Source: PLoS One. 2016 Oct 11;11(10):e0164533. doi: 10.1371/journal.pone.0164533 (PMC5058512; doi:10.1371/journal.pone.0164533)
Supplement: S7 Fig — NMDS ordination plots (Bray-Curtis similarity) of (A) bacterial and (B) fungal community structures colonizing Brachypodium seminal and nodal root tips and bases at day 30 and day 44 after sowing. Bacterial 16S rRNA and fungal ITS gene diversities were analyzed with T-RFLP. Data are mean NMDS scores for axes 1 and 2 ± SE (n = 9). (PDF) [file pone.0164533.s007.pdf]

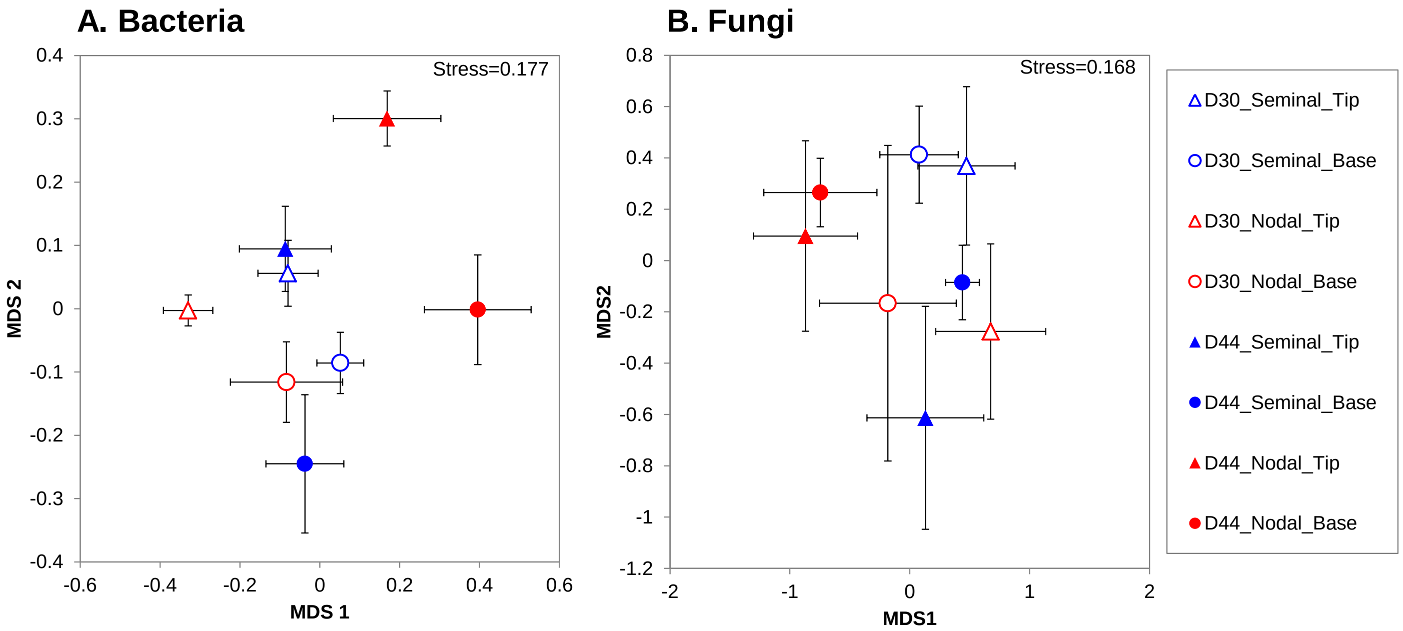

S7 Fig. Microbial community structures of Brachypodium roots. NMDS ordination plots (Bray-Curtis similarity) of (A) bacterial and (B) fungal community structures colonizing Brachypodium seminal and nodal root tips and bases at day 30 and day 44 after sowing. Bacterial 16S rRNA and fungal ITS gene diversities were analyzed with T-RFLP. Data are mean NMDS scores for axes 1 and 2 ± SE (n=9).
