# Supplementary material for: Microbiome and Exudates of the Root and Rhizosphere of Brachypodium distachyon, a Model for Wheat
Source: PLoS One. 2016 Oct 11;11(10):e0164533. doi: 10.1371/journal.pone.0164533 (PMC5058512; doi:10.1371/journal.pone.0164533)
Supplement: S10 Fig — Bacterial community was analyzed with 16S pyrosequencing, and 3534 sequences were randomly subsampled from each sample to achieve even sequencing depth. Means are shown ± SE (n = 8–9). (PDF) [file pone.0164533.s010.pdf]

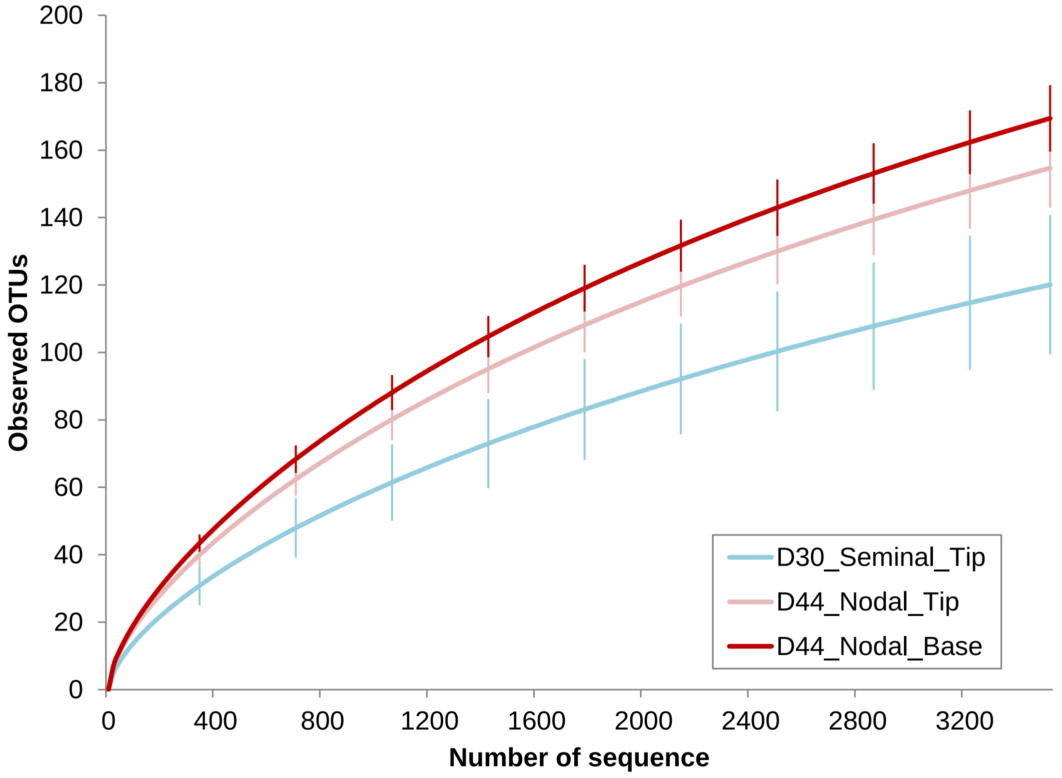

S10 Fig. Rarefaction curves of bacterial OTUs identified in Brachypodium seminal and nodal root systems. Bacterial community was analyzed with 16S pyrosequencing, and 3534 sequences were randomly subsampled from each sample to achieve even sequencing depth. Means are shown ± SE (n=8-9).
